# Comprehensive Analysis of Copy Number Variation of Genes at Chromosome 1 and 10 Loci Associated with Late Age Related Macular Degeneration

**DOI:** 10.1371/journal.pone.0035255

**Published:** 2012-04-25

**Authors:** Stuart Cantsilieris, Stefan J. White, Andrea J. Richardson, Robyn H. Guymer, Paul N. Baird

**Affiliations:** 1 Centre for Eye Research Australia, University of Melbourne, Royal Victorian Eye and Ear Hospital, East Melbourne, Victoria, Australia; 2 Centre for Reproduction and Development, Monash Institute of Medical Research, Melbourne, Victoria, Australia; Radboud University Nijmegen Medical Centre, The Netherlands

## Abstract

Copy Number Variants (CNVs) are now recognized as playing a significant role in complex disease etiology. Age-related macular degeneration (AMD) is the most common cause of irreversible vision loss in the western world. While a number of genes and environmental factors have been associated with both risk and protection in AMD, the role of CNVs has remained largely unexplored. We analyzed the two major AMD risk-associated regions on chromosome 1q32 and 10q26 for CNVs using Multiplex Ligation-dependant Probe Amplification. The analysis targeted nine genes in these two key regions, including the Complement Factor H (*CFH*) gene, the 5 CFH-related (*CFHR*) genes representing a known copy number “hotspot”, the *F13B* gene as well as the *ARMS2* and *HTRA1* genes in 387 cases of late AMD and 327 controls. No copy number variation was detected at the *ARMS2* and *HTRA1* genes in the chromosome 10 region, nor for the *CFH* and *F13B* genes at the chromosome 1 region. However, significant association was identified for the *CFHR3-1* deletion in AMD cases (p = 2.38×10^−12^) OR = 0.31, CI-0.95 (0.23–0.44), for both neovascular disease (nAMD) (p = 8.3×10^−9^) OR = 0.36 CI-0.95 (0.25–0.52) and geographic atrophy (GA) (p = 1.5×10^−6^) OR = 0.36 CI-0.95 (0.25–0.52) compared to controls. In addition, a significant association with deletion of *CFHR1-4* was identified only in patients who presented with bilateral GA (p = 0.02) (OR = 7.6 CI-0.95 1.38–41.8). This is the first report of a phenotype specific association of a CNV for a major subtype of AMD and potentially allows for pre-diagnostic identification of individuals most likely to proceed to this end stage of disease.

## Introduction

Age-related Macular Degeneration (AMD) is a complex disease associated with multiple gene and environment interactions [1]. It is also the leading cause of vision loss in the western world and ranks third among global causes of visual impairment [2]. Rapid progress in identifying genetic risk factors for AMD susceptibility has been made over the last few years including the identification of two major loci at chromosome 1q32 and 10q26. Whilst these two loci appear to account for the majority of genetic susceptibility in Caucasian populations with AMD, the mechanisms behind their involvement in AMD development and progression are still incompletely understood [3].

Copy Number Variations (CNVs) (deletions and duplications >1000 bp) have recently been shown to have an important role in complex disease phenotypes, most notably in autoimmune related disorders such as Psoriasis [4], Rheumatoid Arthritis [5], Systemic Lupus Erythematosus (SLE) [6] and Atypical Hemolytic Uremic Syndrome (aHUS) [7]. A great proportion of CNVs are enriched towards secreted, olfactory and immunity proteins [8] and it is well known that regions flanked by duplicons (regions of high sequence similarity >95% and a at least 10kb in length) are “hotspots” for CNV and thus more likely to undergo recurrent rearrangements due to non-allelic homologous recombination (NAHR) [9].

Analysis of the Regulators of Complement Activation (RCA) alpha block located on chromosome 1q32 that contain the *CFH*, *CFH* related 1–5 and *F13B* genes and analysis of the chromosome 10q26 region containing the *ARMS2* and *HTRA1* genes has identified a series of single nucleotide polymorphisms (SNPs) associated with both risk of, and protection against, AMD [10], [11]. It has previously been shown that an 84 kb deletion encompassing the entire *CFHR*3 and *CFHR*1 genes is associated with protection in AMD [12], [13]. This same deletion is inversely associated with aHUS, thus representing a significant genetic risk factor [14]. Of the five ancestrally related *CFHR* genes, *CFHR3*, *CFHR1* and *CFHR4* are flanked by segmental duplicons and re-arrangements between these duplicons are common but vary in frequency between ethnicities [15]. At the chromosome 10 locus, the two genes, *ARMS2* and *HTRA1* have been extensively screened using SNPs and inferred SNP haplotypes [11], [16], but these genes have yet to be analyzed for larger structural variation which may impact on disease pathogenesis. Direct sequencing of this region in a small number of individuals has already revealed an insertion/deletion (indel) polymorphism in the 3′UTR (del443ins54) of *AMRS2*, showing significant association with increased risk of AMD [17].

On the basis of the genomic architecture of the RCA block and the as yet unexplored chromosome 10 regions for CNV, we hypothesized that, in addition to the common *CFHR3-1* deletion, other potential rearrangements encompassing the *CFH, CFHR4, CFHR2, CFHR5, F13B, ARMS2 and HTRA1* genes may contribute to AMD pathogenesis. Two recent reports of a rare *CFHR1-4* deletion further support this notion that other CNVs exist in these regions [18], [19]. To assess this region in more detail we have applied Two-colour Multiplex Ligation-dependant Probe Amplification (MLPA), a well established method for detecting quantitative changes in DNA [20], [21] and quantitative polymerase chain reaction (qPCR) to validate our findings in a cohort of AMD cases and controls from Australia.

## Materials and Methods

### Study Design

The study was conducted in accordance with the Declaration of Helsinki and according to the National Health and Medical Research Council of Australia's statement on ethical conduct in research involving humans, revised in 2000. Written informed consent was obtained from all individuals, and ethics approval for the project was provided by the Human Research and Ethics Committee of the Royal Victorian Eye and Ear Hospital (RVEEH), Melbourne. The detailed methods of clinical diagnosis of late AMD as well as control samples has been previously reported [22]. In brief, this was a prospective study with all patients recruited from the RVEEH upon attendance at the clinic. All individuals included in this study were Caucasian of Anglo-Celtic ethnic background. At the time of recruitment, a standard risk factor and disease history questionnaire was undertaken, a clinical examination was performed, a fundus photograph obtained, and a blood sample collected for DNA analysis. Cases had a diagnosis of late AMD which consisted of either neovascular disease (nAMD) or geographic atrophy (GA) whereas control individuals were included if they had a normal fundus (<10 hard drusen <63 µm in size) and no altered macular pigmentation in either eyes. 268 cases on nAMD, 86 Bilateral GA, 33 unilateral GA and 327 normal controls were utilized for this study (Table 1). Cases of nAMD were verified in the clinic on angiography or in clinical notes from the treating clinician. Cases and controls were graded using the modified International Classification System for AMD by two independent graders.

**Table 1:** Characteristics of the study population: Age and Gender Distribution of AMD Cases and Controls.

	Unilateral GA (n = 33)	nAMD (n = 268)	Bilateral Geographic Atrophy (n = 86)	Total Cases (n = 387)	Controls (n = 327)
Female n (%)	15 (45)	173 (64)	59 (68)	248 (64)	182 (47)
Age (SD)	69±(10.2)	76±(7.7)	71±(9.6)	74±(8.6)	71±(7)

### Multiplex Ligation-dependant Probe Amplification (MLPA)

#### Probe Design

Two-colour MLPA as described by White *et al.* [21] was used to assess CNVs. Probes were designed to each coding exon of *HTRA1* (9 exons) and *ARMS2* (2 exons), 17 of the 23 coding exons of *CFH* and 3 coding exons contained within *F13B*. To analyze the *CFHR1-5* region 5 probes were positioned within the 2 flanking segmental duplicons extending from *CFHR3 to CFHR4*, while another 4 probes were designed within the remaining *CFHR2* and *CFHR5* genes (Table S1). To allow for simultaneous probe amplification, the common ends of each probe corresponded to the MLPA primers described by Schouten *et al.* [20] and the Multiplex Amplifiable Probe Hybridization (MAPH) primers described by White *et al.* [23]. The specificity of the probes was analyzed using the BLAT program at the University of California Santa Cruz (http://genome.ucsc.edu). Probes were designed to produce PCR products ranging from 80 base pairs (bp) to 130 bp. Oligonucleotides were ordered from Sigma Genosys (www.sigma.genosys.com) with the 5′ end of the right hand half-probe phosphorylated to allow ligation to occur. Probe mixes were prepared by combining each oligonucleotide so that they were all present to a final concentration of 4 fmol/µl.

#### MLPA Reaction

Reagents for the MLPA reaction were purchased from Fisher Biotec (Australia) with the exception of MAPH primers that were purchased from Sigma Genosys. The MLPA reactions were performed according to the method published by Schouten *et al.* [20]. Briefly, 250 ng of DNA in a final volume of 5 µl was denatured at 98°C for 5 minutes, after cooling at room temperature, 1.5 µl of probe mix and 1.5 µl of Hybridization buffer was added to the sample, heat denatured at 95°C for 1 minute followed by hybridization at 60°C for 16 hours.

Ligation was performed at 54°C by adding 32 µl of ligation reaction, after 15 minutes the enzyme was inactivated by heating at 95°C for 5 minutes.

Polymerase Chain Reaction (PCR) amplification was carried out under the following conditions: 1 cycle of 98°C for 1 minute; 35 cycles of 95°C 30 seconds, 57°C 1 minute, 72°C 1 minute; and 1 cycle of 72°C 20 minutes. The PCR reaction was performed in 25 µl.

From each PCR reaction, 1 µl was mixed with 8.8 µl of HIDI formamide and 0.2 µl of LIZ500 size standard (Applied Biosystems). Product separation was performed on the ABI 3130 Electrophoresis 16 capillary sequencer (Applied Biosystems).

#### Data Analysis

Data Analysis was performed using the GeneMapper Software (Applied Biosystems) and the peak heights were exported to Microsoft Excel. A “global” or “population based” analysis was implemented whereby normalization was performed by calculating the sum of all peak heights from each sample and dividing by the individual peak heights of each probe [24]. The median was calculated for each probe which was then divided by each individual probe to calculate the number of copies of each exon. Thresholds for deletions and duplications were set at below 0.75 and above 1.25 respectively. All samples were tested at least twice.

### Quantitative PCR (qPCR)

To verify the results of MLPA, a qPCR assay was designed for the *CFHR3* and *CFHR4* genes using *FOXP2* as the control gene [25]. The PCR primers for qPCR are listed in Table S2. qPCR was conducted in triplicate using 25 ng of DNA, PCR reactions were performed using 7.5 µl of 2× Bioline Syber Green Master Mix (www.bioline.com), 0.5 µl of 10 pmol/µl primers and 5 µl of DNA in a 15 µl reaction. qPCR cycling protocol consisted of 95°C for 3 minutes; 40 cycles of 95°C for 5 seconds, 63°C for 5 seconds, 72°C for 10 seconds. Copy number calculations were performed using the Delta-Delta CT method and average copy number was set at 2 [25].

### Quantitative Multiplex PCR of Short Fluorescent Fragments (QMPSF)

To verify results of *CFH* exon 18 we employed QMPSF analysis as described by Casili *et al.* [26]. Briefly, primers were designed to flank *CFH* exon 18. The primers used were chimeric and had 5′ extensions of CGTTAGATAG on the forward primer or GATAGGGTTA on the reverse primer. All forward primers were 5′ labeled with 6-FAM. An additional primer pair amplifying the HMBS gene located on chromosome 11 was used as an internal control as described by Saugier-Veber *et al.* [27]. Primer Pairs are listed in the Table S4, PCR reactions were performed as previously described using a modified PCR protocol to allow for quantitative conditions [27].

### Statistics

Chi Square analysis, Fisher's exact test and deviations from Hardy-Weinberg Equilibrium (HWE) was performed using PLINK software v1.07 [28]. All genotypes were in HWE (p = >0.001). Logistic regression models were constructed to determine the odds ratio (OR) and 95% confidence intervals (CI 0.95) for AMD cases while conditioning on the rs1061170 SNP in the *CFH* gene, the rs10490924 SNP in the *LOC387715* gene and the rs11200638 SNP in the *HTRA1* gene after adjustment for age, gender and smoking. Logistic regression and conditional analysis was performed using PLINK v1.07.

## Results

### Patient Samples

A total of 714 individuals consisting of 387 late stage AMD cases with either nAMD (n = 268) or bilateral GA (n = 86) or unilateral GA (n = 33) and controls (n = 327) without AMD were included in the CNV analysis. The average age of all cases at examination was 74 years with those having nAMD having a mean age of 75 years and those having GA a mean age of 71 years. The average age of control participants was 71.4 years. Male to female ratios were 247/136 (64% vs 36%) in cases and 182/145 (53% vs 47%) in controls.

### Probe Design

**Figure 1:**
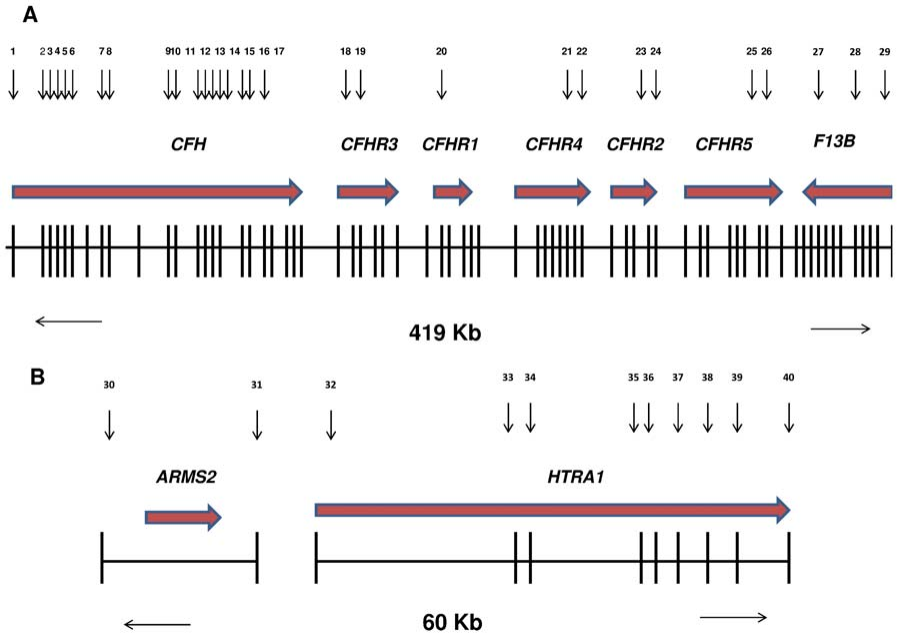
Schematic Representation of Chromosome 1q32 and Chromosome 10q26. (A) Chromosome 1q32 showing a 419 kb region containing the *CFH*, *CFHR1-5* and *F13B* genes. The vertical lines represent exons in each of the genes. The horizontal arrows at the top represent the direction of transcription. The numbers (top) represent the MLPA probe positions across each of the genes. (B) Chromosome 10q26 show a 60 kb region containing the *ARMS2* and *HTRA1* genes. The horizontal arrows at the top represent the direction of transcription. The numbers represent the MLPA probe positioning in each of the coding exons (vertical bars) across these genes.

Copy number analysis was performed on genes from two well established AMD associated genomic loci, targeting seven genes on chromosome 1q32 (*CFH*, *CFHR1*, *CFHR2*, *CFHR3*, *CFHR4*, *CFHR5*, *F13B*) and two genes on chromosome 10q26 (*ARMS2 and HTRA1*) (Figure 1). The probe coverage on chromosome 1 extended almost 0.5 mega base (Mb) from exon 1 of the *CFH* gene to the final exon of the *F13B* gene, while on chromosome 10 the genomic coverage was 60 kilo bases (kb) extending from the first exon of the *ARMS2* gene to the final exon of the *HTRA1* gene. Not all coding exons within the *CFH* gene were amenable to MLPA probe design due to the extensive homology existing between the *CFH* and the *CFHR1-5* genes. Reliable MLPA probe designs could not be achieved for exon 7 and exons 20–23 of the *CFH* gene, but nevertheless we were able to design probes for the majority of the *CFH* gene covering 17/22 its exons. Reliability of probe designs was calculated by assessing the standard deviation (SD) of the normalized probe ratio (1.0 indicating CN of 2) for all 43 probes in 50 samples. The SD for all 43 probes was <10% indicating reliable performance of all probe designs (Table S1).

### Detection of re-arrangements on 1q32 and 10q26

**Figure 2:**
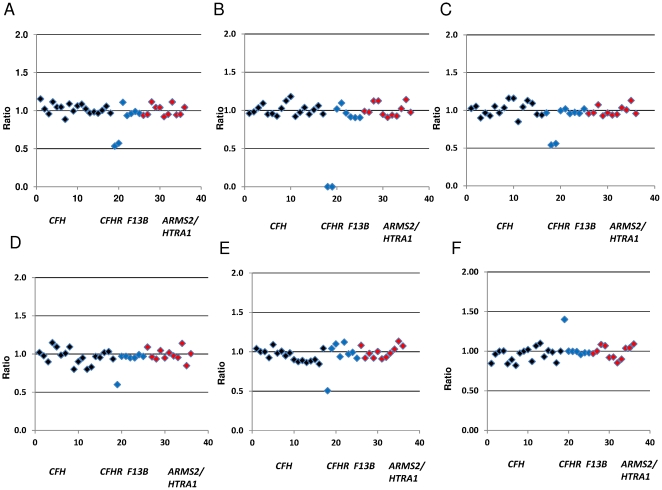
Peak Patterns and Scatter Plots. Scatter plots representing 5 different CNV re-arrangements detected in the *CFHR3*, *CFHR1* and *CFHR4* genes in our sample cohort. Probes targeting the *CFH* gene are represented in black, probes targeting the *CFHR1-5* and *F13B* genes are represented in blue and probes corresponding to the *ARMS2* and *HTRA1* genes are represented in red. The x axis describes each probe number which corresponds to the numbered probe list in Table S1. The y axis represents normalized probe ratios relative to a two copy locus (Normal range 0.75–1.25). The scatter plots describe (A) a heterozygous deletion of *CFHR3-1*, (B) a homozygous deletion of *CFHR3-1*, (C) a heterozygous deletion of *CFHR1-4*, (D) a heterozygous deletion of *CFHR3*, (E) heterozygous deletion of *CFHR1* (F) heterozygous duplication of *CFHR1*.

MLPA analysis did not reveal the presence of any large re-arrangements encompassing either the *ARMS2* or *HTRA1* genes at chromosome 10q26 in either cases or controls. Similarly the *CFH*, *CFHR2*, *CFHR5* and *F13B* genes did not show evidence of CNVs. Analysis of *CFH* exon 18, appeared to indicate the presence of a heterozygous deletion. Further analysis of the ligation site used to detect this deletion revealed a SNP (rs35292876) identified from recent 1000 genomes data (http://genome.ucsc.edu) likely leading to disturbance of the ligation site and thus giving the appearance of a heterozygous deletion. Confirmation that the exon was indeed only present as two copies was made using Quantitative Multiplex PCR of Short Fluorescent fragments (QMPSF) where primers were designed that flanked the exon (Table S4). Within the remaining *CFHR3*, *CFHR1* and *CFHR4* genes, we detected 6 different combinations of re-arrangements, the common *CFHR3-1* deletion (homozygous and heterozygous), the *CFHR1-4* deletion, deletion of *CFHR1* with reciprocal duplication as well as deletion of *CFHR3* (Figure 2).

### Analysis of CNV's and late AMD

Analysis of the common *CFHR3-1* deletion demonstrated its association with protection in AMD being present in a total of 32.8% of controls compared to 13.75% of cases (p = 2.38×10^−12^) OR = 0.31, CI-0.95 (0.23–0.44) (Table 2). The homozygous deletion was almost 10 fold more frequent in controls (5.8%) compared to cases (0.75%), whereas in the heterozygous state it was 2 fold more frequent in controls (27%) compared to cases (13%). Subtype analysis in both end stage nAMD and GA showed that the deletion was protective in both subtypes at (p = 8.3×10^−9^) OR = 0.36 CI-0.95 (0.25–0.52) and (p = 1.5×10^−6^) OR = 0.36 CI-0.95 (0.25–0.52) (Table 2). We performed logistic regression analysis adjusting for gender, age, smoking and conditioning on the Y402H (rs1061170) polymorphism of the *CFH* gene. A statistically significant association remained after adjusting for these confounders (1.34×10^−5^) OR = 0.40 CI-0.95 (0.27–0.60) (Table 3), consistent with previous reports [19], [29], [30]. We also conditioned on other *CFH* SNPs rs800292 and rs1061147 and found the same result (data not shown). Conditional analysis on two of the high risk SNPs in the chromosome 10 region rs10490924 and rs11200638 also showed a statistically significant association with protection from AMD (3.2×10^−9^) OR = 0.28 CI 0.95 (0.19–0.43) (Table 3). Analysis of the rare *CFHR1-4* deletion showed that while the deletion was not associated with risk or protection in late AMD cases as a whole (p = 0.46), stratification by subtype revealed a statistically significant association with risk of cases with bilateral GA (p = 0.02) OR = 7.6 CI 0.95 (1.38–41.8). Analysis in 327 controls showed that the frequency of the deletion was below <1% compared to 4.7% in bilateral GA. We observed no significant difference in the nAMD subtype (p = 1.0). The other three re-arrangements detected in the *CFHR* genes were too rare to infer any statistical significance, consistent with previous reports [31].

**Table 2:** Frequencies of Copy Number Rearrangements and Association with end stage AMD.

			End stage AMD						Unadjusted Analysis					
	Controls (n = 327)		Unilateral GA (n = 33)		Bilateral GA (n = 86)		nAMD(n = 268)		Bilateral GA		nAMD		ALL AMD	
	n	%	n	%	n	%	n	%	OR (95% CI)	P Value	OR (95% CI)	P Value	OR (95% CI)	P Value
**CFHR3-1 Deletion**														
2 Copies	220	67.3	27	82	80	93	229	85.45	0.36 (0.25–0.52)	1.5×10^−6^	0.36 (0.25–0.52)	8.3×10^−9^	0.31 (0.23–0.44)	2.38×10^−12^
1 Copy	88	26.9	6	18	6	7	37	13.8						
0 Copies	19	5.8	0	0	0	0	2	0.75						
**CFHR1-4 Deletion**														
2 Copies	325	99.4	33	100	82	95.3	266	99.26	7.6 (1.38–41.8)	0.02	0.6 (0.05–6.8)	1	2.1 (0.40–10.91)	0.46
1 Copy	2	0.006	0	0	4	4.7	2	0.74						
0 Copies	0	0	0	0	0	0	0	0						

**Table 3:** Logistic regression analysis of rs1061170, rs10490924, rs11200638 and CFHR3-1 deletion in AMD Cases and Controls.

	Bilateral GA		nAMD		ALL AMD	
CFHR3-1 Deletion	OR (95% CI)	P Value	OR (95% CI)	P Value	OR (95% CI)	P Value
Adjusted for Age, Gender, Smoking	0.16 (0.07–0.37)	2.2×10^−5^	0.32 (0.21–0.48)	5.1×10^−8^	0.30 (0.20–0.43)	3.04×10^−10^
Adjusted for Age, Gender, smoking and conditioned on rs1061170	0.24 (0.09–0.62)	0.003	0.42 (0.27–0.64)	8.4×10^−5^	0.40 (0.27–0.60)	1.37×10^−5^
Adjusted for Age, Gender, smoking and conditioned on rs10490924, rs11200638	0.17 (0.07–0.40)	6.6×10^−5^	0.32 (0.21–0.50)	4.4×10^−7^	0.28 (0.19–0.43)	3.2×10^−9^

## Discussion

We have carried out a comprehensive analysis of copy number variation in nine genes from the two most significantly AMD risk associated loci. Identification of the rare heterozygous *CFHR1-4* deletion appears significantly associated with risk of bilateral GA, being 6 times more frequent in this late phenotype compared to that of nAMD. Interestingly this deletion is present in only 0.74% of nAMD cases, but 4.7% in bilateral GA, suggesting that the deletion may contribute to a different pathophysiological process associated with the bilateral GA phenotype (p = 0.03). We also provide further evidence for the protective role of the *CFHR3-1* deletion in AMD, with the homozygous deletion being present in 5.4% of control samples compared to only 0.75% of cases. Three other rare re-arrangements in *CFHR1* and *CFHR3* were also identified in two control samples and one case including a heterozygous deletion of *CFHR3* and a heterozygous deletion and a duplication of *CFHR1*. Analysis of the coding exons of the *CFH* gene, did not reveal any contiguous or non-contiguous CNVs extending from the *CFHR* region. Similarly, analysis of the *F13B* gene in a subset of 100 cases and 100 controls did not show evidence of CNVs. The *ARMS2* and *HTRA1* genes also showed no evidence of CNVs, suggesting that the *ARMS2* indel (del443ins54) located in the promoter region may represent the only major and relatively large structural variant at this locus.

Population based frequencies of the *CFHR3-1* and *CFHR1-4* deletions show evidence of population stratification across multiple ethnicities [12], [32]. A study by Hageman *et al.* showed that the frequency of the homozygous *CFHR3-1* deletion in Africans was as high as 16% compared to 7% in Hispanics, 5% in Caucasians, and only 2% in Asians [12]. Data from the 1000 genomes project [32] also supports this finding indicating that combined analysis of heterozygous and homozygous *CFHR3-1* deletions are present in approximately 50% of Africans compared to 25% in Europeans and less that 10% in Asians [32]. The European frequency is comparable to the combined analysis of homozygous and heterozygous deletions at 32.8% reported here [12], [18]. The *CFHR3-1* deletion frequency within African populations may also account for the reduced prevalence of AMD within this population compared to Europeans [33] although it cannot be ruled out that this invariably reflects an earlier age of mortality in this group. Nevertheless it cannot be discounted that the presence of this CNV is positively selected given that these genes have recently been identified as functionally important in complement regulation [29]. Analysis of the rare *CFHR1-4* deletion in our Caucasian control population showed the frequency to be <1%. This frequency was similar to data from the 1000 genomes project which showed no evidence of this CNV in Caucasians and below 5% frequency in Africans and Asians (n = 159) [32].

Analysis of the *CFHR3-1* deletion in our study found similar frequencies to those described previously of homozygous deletions in cases (0.8%–1.2%) and controls (4.9%–5.2%), similarly heterozygous deletions between cases (17%–18%) and controls (22%–27%) [12], [31]. Like others [12], [13], we also identified a clear gene dosage effect between *CFHR3-1* heterozygous and homozygous deletion samples where individuals with homozygote deletions conferred higher protection from AMD. A study by Fritsche *et al.* demonstrated that homeostatic balance between *CFHR1*, *CFHR3* and *CFH* determines complement activity and influences inflammation [29]. *CFHR3* and *CFHR1* compete with *CFH* for C3b binding, the loss of two complement regulators from the homozygous deletion of *CFHR3/1* results in increased binding of *CFH to C3b* thereby regulating *CFH* mediated complement activity [29]. They also showed this affect was independent of the Y402H and A473A polymorphism of the *CFH* gene further confirming that this CNV is functionally relevant in AMD pathogenesis. Consistent with these findings and others [19], [29], [30], we also showed that this effect is independent of Y402H. Studies attempting to verify the independent effects of this deletion have shown that while the statistical strength is mitigated upon conditioning of several highly associated SNPs within *CFH*, the statistical significance is not entirely removed [29], [30]. A recent report by Hughes *et al.* suggested that the reduction in P value, was a reflection on allele frequency, rather than affect size, as the OR's between rs2274700 and rs10737680 confer almost equal effects to that of the *CFHR3-1* deletion (0.37–0.39) [34].

While this manuscript was in preparation three other studies reported on CNVs in the *CFHR1-5* region with AMD [18], [19], [35]. Sivakumaran *et al.* [35] reported that an increase *CFHR3-1* CN showed trends towards risk, before conditioning on the most significant SNPs in *CFH.* Interestingly, we and others [12], [13], [18], [29], [31] have not detected enrichment of increased *CFHR3-1* copy numbers in AMD cohorts, in fact this appears to be a rare event. In contrast to our data, and others, Sivakumaran *et al.* also reported that additional copies of *CFHR1-4* appeared to confer risk to AMD, suggesting that the deletion conferred protection [35]. The reciprocal duplication of *CFHR1-4* appears to also be a rare event in Africans, Asians and European control populations [32], and was not detected in two subsequent studies of AMD cases and controls [18], [19]. This may be a reflection of ethnic differences between the cohorts or the methods used to genotype the re-arrangements at the *CFHR1-5* region. Several rare re-arrangements, the majority of which, residing in intergenic regions between the *CFHR1-5* genes has been reported [35]. *De-novo* events affecting several exons within *CFH* were detected within samples from African ancestry. In support of our data, these changes were not detected among Caucasian samples suggesting that re-arrangements within *CFH* are rare events, unlikely to have an impact on AMD pathogenesis in individuals of European origin [35].

While there appears to be overwhelming consensus in relation to the association of the *CFHR3-1* deletion and protection from AMD, the same cannot be said for the rare CNV of *CFHR1-4.* Two recent studies found no significant association with this variant and AMD [18], [19]. The frequency of this deletion appears to fluctuate depending on the cohort and methodologies used for genotyping. In addition, the deletion and or reciprocal duplication have not been found to be in linkage disequilibrium (LD) with flanking SNP markers [18], [35], thus must be genotyped directly. Both studies by Sawitzke *et al.* and Sivakumaran *et al.* used samples from the Age Related Eye Disease Study (AREDS) but appeared to yield different frequencies of *CFHR1-4* copy numbers [19], [35]. These findings of differing copy number changes in two sub populations of AREDS individuals cannot be easily explained. One explanation is the potential for admixture in the population and it would be interesting if the two sub cohorts were assessed for this effect. Ethnicity information was not reported in either of the Sawitzke or Sivakumaran studies. Another potential explanation is that two differing methodologies used to analyze this region (qPCR and array CGH) may have led to some methodological biases between the two studies [19], [35].

Interestingly, several studies of CNVs in other diseases have shown similar conflicting data. Lower copy number of the FC Gamma Receptor 3B (*FCGR3B*) gene has been shown to be associated with Rheumatoid Arthritis (RA) [5], while other studies have failed to replicate this association [25], [36]. Similarly, analysis of CC Chemokine Ligand 3-like 1 (*CCL3L1*) in HIV AIDS susceptibility has found evidence of lower copy number and HIV susceptibility [37], while others have found no association [38], [39]. It remains unclear as to whether accurate assessment of CNVs, population sub structure, admixture, or whether small changes in detected frequencies of rare CNVs in cases and controls affected by sample size are the main contributing factors to the reproducibility of these associations.

Functionally the *CFHR4* gene has been shown to be important in complement regulation [40]. *CFHR4* protein binds to a pentameric form of C-reactive protein (pCRP) (different from the monomeric *CRP* bound by *CFH*) and recruits pCRP to the surface of necrotic cells enhancing removal of necrotic cells directly or facilitating C1q binding and complement activation [41]. In addition, it has been proposed that *CFHR4* limits inflammation by enhancing the co-factor activity of *CFH* and enhancing complement deposition by CRP binding which helps phagocytic clearance of microbes and necrotic cells in inflamed tissues [40]. If *CFHR4* regulates complement activation and opsonization on biological surfaces via interaction with pCRP [40], then potentially a deletion of this gene would lead to reduced pCRP binding and thus limit its capacity to inhibit inflammation leading to enhanced disease. A similar hypothesis has also been suggested for the *CFH* gene by Laine *et al.* [42] whereby binding affinity of CRP for *CFH* was influenced by the Y402H polymorphism. It was suggested that impaired binding of CRP to *CFH* reduced the ability for *CFH* to modulate inflammation [42]. Furthermore, no individuals in the current study presented with both the *CFHR3-1* and *CFHR1-4* deletions on both alleles as described previously [14]. As these genes represent a functionally different component to complement regulation, it suggests that homeostatic balance between *CFH*, *CFHR3*, *CFHR1* and *CFHR4* is necessary to maintain regulation of complement activation.

Typically, studies analyzing the *CFHR1-5* gene cluster have incorporated an MLPA based approach [13], [14], [18], [29], [31]. MLPA utilizes a ligation step to join two adjacent oligonucleotide probe sequences together after hybridization [20]. This step provides increased sensitivity to distinguish between two highly homologous sequences [43]. However, an unsuspected polymorphism near the ligation site of the two MLPA oligonucleotides can hamper accurate determination of CNV regions [44]. In most primer probe based assays, a polymorphism can disturb primer binding enough to give the appearance of a deletion [45]. In this case a deletion detected with a single probe should always be confirmed via an alternative method. With this in mind we implemented three methods of CNV detection through the use of MLPA, QMPSF and qPCR.

Data from Kubista *et al.* previously identified a heterozygous deletion of *CFHR2* at a frequency of less than 1% [18]. We and others [19], [35] did not detect this deletion in our respective Caucasian cohorts. One possible explanation is a potential polymorphism on the ligation site for the *CFHR2* MLPA probe used by Kubista *et al*. [18]. Sequence analysis revealed a polymorphism C>T (rs72736421) directly on the ligation site which is likely to disturb the probe binding sufficiently to give the appearance of a heterozygous deletion. We identified similar polymorphisms for probes used to analyze *CFH* exon 18 (rs115722139) and *CFH* intron 1 (rs77837548), although these polymorphisms were further away from the ligation site, 8 bp and 3 bp respectively [18]. In the case of the *CFH* exon 18 MLPA probe used in this study, analysis of the ligation site showed a polymorphism C>T (rs35292876) directly on the ligation site which would explain the presence of an apparent deletion that was initially detected using this technique but yet could not be detected in confirmatory studies using QMPSF (Table S3). The polymorphism was not identified during the original probe design and was only identified following release of the first draft of the 1000 genomes data.

Our understanding of CNVs and the role that they play in complex disease is likely to increase substantially in the next few years, especially with recent advancements in next generation sequencing and array based Comparative Genome Hybridization [46], [47]. Of continuing interest is the apparent reversal of associations seen in seemingly unrelated complex diseases to what are essentially identical copy number variants. For example, studies of the RCA alpha block encompassing *CFH* and *CFHR1-5* genes have shown associations with AMD, aHUS, Membranoproliferative Glomerulonephritis [48] and SLE. Curiously, the *CFHR3-1* deletion has now been shown to be associated with risk in aHUS [14] and SLE [49] but protection in AMD [12]. The same study in aHUS individuals also showed that combined deletion of *CFHR3-1* and *CFHR1-4* on both alleles is associated with risk of aHUS [14]. Similarly studies analyzing the beta defensin gene cluster have shown that while increased beta defensin copy number (>5 copies) is associated with psoriasis, low copy numbers (<4 copies) are associated with Crohn's Disease [50], [51]. Further investigation into the functionality of these CNV regions is required to assess their impact on complex disease pathogenesis. These findings may indicate a dosage response mechanism or differing action depending on the tissue type.

Our finding that the rare *CFHR1-4* deletion is associated with risk of bilateral GA may have important research and clinical implications, particularly as our current knowledge regarding how progression of AMD occurs towards the two late stage phenotypes of GA and nAMD is currently limited. While this is an interesting finding, we are cautious about reporting associations of rare variants especially given the conflicting data from three other reports. Replication in larger populations would strengthen the argument that the findings reported here are reproducible before any definitive conclusions can be drawn concerning a clinically relevant association between this variant and AMD.

Our data represents a comprehensive study of copy number variation in genes associated with AMD. Our cohort was collected from a single centre with all cases being seen by a small number of retinal specialists. The phenotype information was well characterized with ethnicity information collected. In summary we demonstrate that the relevant copy number “hotspot” in AMD lies in the region encompassing the *CFHR3*, *CFHR1* and *CFHR4* genes. These findings support the idea that CNVs do impart a likely functional role in AMD pathogenesis through gene dosage effects on genes regulating the complement cascade. Given that many copy number polymorphisms (CNPs) are poorly tagged by SNPs, and are likely to exert their own independent effects, a combined effort analyzing all classes of genetic variation such as SNPs, Indels and CNVs is likely to lead to a greater understanding of AMD pathogenesis. Given the substantial number of people affected by AMD this finding if replicated would greatly enhance our ability to predict which patients are most likely to go on to develop GA and thereby offer specific treatments targeted to such a group.

## Supporting Information

Table S1 MLPA Probe Sequences with Corresponding Standard Deviation values for each probe.
(DOC)

Table S2 Real time Quantitative PCR primer sequences.
(DOC)

Table S3 Validation of copy number changes in the *CFH* gene using locus specific QMPSF.
(DOC)

Table S4 Primers sequences for QMPSF analysis.
(DOC)

## References

[1] Guymer RH, Chong EWT (2006). Modifiable risk factors for age-related macular degeneration.. Med J Aust.

[2] Resnikoff S, Pascolini D, Mariotti SP, Pokharel GP (2008). Global magnitude of visual impairment caused by uncorrected refractive errors in 2004.. Bull World Health Organ.

[3] Robman L, Baird PN, Dimitrov PN, Richardson AJ, Guymer RH (2010). C-Reactive Protein Levels and Complement Factor H Polymorphism Interaction in Age-related Macular Degeneration and Its Progression.. Ophthalmology.

[4] de Cid R, Riveira-Munoz E, Zeeuwen P, Robarge J, Liao W (2009). Deletion of the late cornified envelope LCE3B and LCE3C genes as a susceptibility factor for psoriasis.. Nat Genet.

[5] McKinney C, Fanciulli M, Merriman ME, Phipps-Green A, Alizadeh BZ (2010). Association of variation in Fc gamma receptor 3B gene copy number with rheumatoid arthritis in Caucasian samples.. Ann Rheum Dis.

[6] Fanciulli M, Norsworthy PJ, Petretto E, Dong R, Harper L (2007). FCGR3B copy number variation is associated with susceptibility to systemic, but not organ-specific, autoimmunity.. Nat Genet.

[7] Zipfel PF, Edey M, Heinen S, Jozsi M, Richter H (2007). Deletion of Complement Factor Related Genes *CFHR1* and *CFHR3* Is Associated with Atypical Hemolytic Uremic Syndrome.. PLoS Genet.

[8] Nguyen D-Q, Webber C, Ponting CP (2006). Bias of selection on human copy-number variants.. PLoS Genet.

[9] Sharp AJ, Locke DP, McGrath SD, Cheng Z, Bailey JA (2005). Segmental duplications and copy-number variation in the human genome.. Am J Hum Genet.

[10] Hageman GS, Anderson DH, Johnson LV, Hancox LS, Taiber AJ (2005). A common haplotype in the complement regulatory gene factor H (HF1/CFH) predisposes individuals to age-related macular degeneration.. Proc Natl Acad Sci U S A.

[11] DeWan A, Liu M, Hartman S, Zhang SS, Liu DT (2006). HTRA1 Promoter Polymorphism in Wet Age-Related Macular Degeneration.. Science.

[12] Hageman GS, Hancox LS, Taiber AJ, Gehrs KM, Anderson DH (2006). Extended haplotypes in the complement factor H (CFH) and CFH-related (CFHR) family of genes protect against age-related macular degeneration: Characterization, ethnic distribution and evolutionary implications.. Ann Med.

[13] Hughes AE, Orr N, Esfandiary H, Diaz-Torres M, Goodship T (2006). A common CFH haplotype, with deletion of CFHR1 and CFHR3, is associated with lower risk of age-related macular degeneration.. Nat Genet.

[14] Moore I, Strain L, Pappworth I, Kavanagh D, Barlow PN (2010). Association of factor H autoantibodies with deletions of CFHR1, CFHR3, CFHR4, and with mutations in CFH, CFI, CD46, and C3 in patients with atypical hemolytic uremic syndrome.. Blood.

[15] Campbell CD, Sampas N, Tsalenko A, Sudmant PH, Kidd JM (2011). Population-Genetic Properties of Differentiated Human Copy-Number Polymorphisms.. Am J Hum Genet.

[16] Atsuhiro K, Wei C, Othman M, Branham KEH, Brooks M (2007). A variant of mitochondrial protein LOC387715/ARMS2, not HTRA1, is strongly associated with age-related macular degeneration.. Proc Nat Acad Sci USA.

[17] Fritsche LG, Loenhardt T, Janssen A, Fisher SA, Rivera A (2008). Age-related macular degeneration is associated with an unstable ARMS2 (LOC387715) mRNA.. Nat Genet.

[18] Kubista KE, Tosakulwong N, Wu Y, Ryu E, Roeder JL (2011). Copy number variation in the complement factor H-related genes and age-related macular degeneration.. Mol Vis.

[19] Sawitzke J, Im KM, Kostiha B, Dean M, Gold B (2011). Association Assessment of Copy Number Polymorphism and Risk of Age-Related Macular Degeneration.. Ophthalmology.

[20] Schouten JP, McElgunn CJ, Waaijer R, Zwijnenburg D, Diepvens F (2002). Relative quantification of 40 nucleic acid sequences by multiplex ligation-dependent probe amplification.. Nucleic Acids Res.

[21] White SJ, Vink GR, Kriek M, Wuyts W, Schouten J (2004). Two-color multiplex ligation-dependent probe amplification: Detecting genomic rearrangements in hereditary multiple exostoses.. Hum Mutat.

[22] Richardson AJ, Islam FMA, Aung KZ, Guymer RH, Baird PN (2010). An Intergenic Region between the tagSNP rs3793917 and rs11200638 in the HTRA1 Gene Indicates Association with Age- Related Macular Degeneration.. Invest Ophthalmol Vis Sci.

[23] White S, Kalf M, Liu Q, Villerius M, Engelsma D (2002). Comprehensive Detection of Genomic Duplications and Deletions in the DMD Gene, by Use of Multiplex Amplifiable Probe Hybridization.. Am J Hum Genet.

[24] White SJ, Breuning MH, den Dunnen JT (2004). Detecting Copy Number Changes in Genomic DNA: MLPA and MAPH.. Methods Cell Biol.

[25] Marques RB, Thabet MM, White SJ, Houwing-Duistermaat JJ, Bakker AM (2010). Genetic Variation of the Fc Gamma Receptor 3B Gene and Association with Rheumatoid Arthritis.. PloS One.

[26] Casilli F, Di Rocco ZC, Gad S, Tournier I, Stoppa-Lyonnet D (2002). Rapid detection of noval BRCA1 rearrangements in high-risk breast-ovarian cancer families using multiplex PCR of short fluorescent fragments.. Hum Mutat.

[27] Saugier-Veber P, Goldenberg A, Drouin-Garraud V, de la Rochebrochard C, Layet V (2006). Simple detection of genomic microdeletions and microduplications using QMPSF in patients with idiopathic mental retardation.. Eur J Hum Genet.

[28] Purcell S, Neale B, Todd-Brown K, Thomas L, Ferreira MAR (2007). PLINK: A Tool Set for Whole-Genome Association and Population-Based Linkage Analyses.. The American Journal of Human Genetics.

[29] Fritsche LG, Lauer N, Hartmann A, Stippa S, Keilhauer CN (2010). An imbalance of human complement regulatory proteins CFHR1, CFHR3 and factor H influences risk for age-related macular degeneration (AMD).. Hum Mol Genet.

[30] Raychaudhuri S, Ripke S, Li M, Neale BM, Fagerness J (2010). Associations of CFHR1-CFHR3 deletion and a CFH SNP to age-related macular degeneration are not independent.. Nat Genet.

[31] Schmid-Kubista KE, Tosakulwong N, Wu Y, Ryu E, Hecker LA (2009). Contribution of Copy Number Variation in the Regulation of Complement Activation Locus to Development of Age-Related Macular Degeneration.. Invest Ophthalmol Vis Sci.

[32] Sudmant PH, Kitzman JO, Antonacci F, Alkan C, Malig M (2010). Diversity of Human Copy Number Variation and Multicopy Genes.. Science.

[33] Klein R, Chou C-F, Klein BEK, Zhang X, Meuer SM (2011). Prevalence of Age-Related Macular Degeneration in the US Population.. Arch Ophthalmol.

[34] Hughes AE, Orr N, Cordell HJ, Goodship T (2010). Reply to Associations of CFHR1-CFHR3 deletion and a CFH SNP to age-related macular degeneration are not independent.. Nat Genet.

[35] Sivakumaran TA, Igo RP, Kidd JM, Itsara A, Kopplin LJ (2011). A 32 kb Critical Region Excluding Y402H in CFH Mediates Risk for Age-Related Macular Degeneration.. Plos One.

[36] Mamtani M, Anaya JM, He W, Ahuja SK (2010). Association of Copy Number Variation in the FCGR3B gene with risk of autoimmune diseases.. Genes Immun.

[37] Gonzalez E, Kulkarni H, Bolivar H, Mangano A, Sanchez R (2005). The Influence of CCL3L1 Gene-Containing Segmental Duplications on HIV-1/AIDS Susceptibility.. Science.

[38] Field SF, Howson JMM, Maier LM, Walker S, Walker NM (2009). Experimental aspects of copy number variant assays at CCL3L1.. Nat Med.

[39] Bhattacharya T, Stanton J, Kim E-Y, Kunstman KJ, Phair JP (2009). CCL3L1 and HIV/AIDS susceptibility.. Nat Med.

[40] Hebecker M, Okemefuna AI, Perkins SJ, Mihlan M, Huber-Lang M (2011). Molecular basis of C-reactive protein binding and modulation of complement activation by factor H-related protein 4.. Mol Immunol.

[41] Mihlan M, Hebecker M, Dahse H-M, Halbich S, Huber-Lang M (2009). Human complement factor H-related protein 4 binds and recruits native pentameric C-reactive protein to necrotic cells.. Mol Immunol.

[42] Laine M, Jarva H, Seitsonen S, Haapasalo K, Lehtinen MJ (2007). Y402H Polymorphism of Complement Factor H Affects Binding Affinity to C-Reactive Protein.. J Immunol.

[43] White S, Vissers L, Geurts Van Kessel A, De Menezes R, Kalay E (2007). Variation of CNV distribution in five different ethnic populations.. Cytogenet Genome Res.

[44] Notini AJ, Craig JM, White SJ (2008). Copy number variation and mosaicism.. Cytogenetic And Genome Research.

[45] Janssen B, Hartmann C, Scholz V, Jauch A, Zschocke J (2005). MLPA analysis for the detection of deletions, duplications and complex rearrangements in the dystrophin gene: potential and pitfalls.. Neurogenetics.

[46] Alkan C, Kidd JM, Marques-Bonet T, Aksay G, Antonacci F (2009). Personalized copy number and segmental duplication maps using next-generation sequencing.. Nat Genet.

[47] Cooper GM, Coe BP, Girirajan S, Rosenfeld JA, Vu TH (2011). A copy number variation morbidity map of developmental delay.. Nat Genet.

[48] Abrera-Abeleda MA, Nishimura C, Smith JLH, Sethi S, McRae JL (2006). Variations in the complement regulatory genes factor H (CFH) and factor H related 5 (CFHR5) are associated with membranoproliferative glomerulonephritis type II (dense deposit disease).. J Med Gen.

[49] Zhao J, Wu H, Khosravi M, Cui H, Qian X (2011). Association of Genetic Variants in Complement Factor H and Factor H-Related Genes with Systemic Lupus Erythematosus Susceptibility.. PLoS Genet.

[50] Fellermann K, Stange DE, Schaeffeler E, Schmalzl H, Wehkamp J (2006). A Chromosome 8 Gene-Cluster Polymorphism with Low Human Beta-Defensin 2 Gene Copy Number Predisposes to Crohn Disease of the Colon.. Am J Hum Genet.

[51] Hollox EJ, Huffmeier U, Zeeuwen P, Palla R, Lascorz J (2008). Psoriasis is associated with increased beta-defensin genomic copy number.. Nat Genet.

