# Supplementary material for: Comprehensive Analysis of Copy Number Variation of Genes at Chromosome 1 and 10 Loci Associated with Late Age Related Macular Degeneration
Source: PLoS One. 2012 Apr 25;7(4):e35255. doi: 10.1371/journal.pone.0035255 (PMC3338825; doi:10.1371/journal.pone.0035255)
Supplement: Table S1 — MLPA Probe Sequences with Corresponding Standard Deviation values for each probe. (DOC) [file pone.0035255.s001.doc]

**Table S1: MLPA Probe Sequences with Corresponding Standard Deviation values for each probe**

| **Probe N#** | **MLPA Probe** | **SD** | **Upstream Hybridising Sequence 5’- 3’** | **Down Stream Hybridising Sequence 5’ - 3’** |
| --- | --- | --- | --- | --- |
| 1 | CFH EXON 1 | 0.07 | CCTGCACTAATCACAATTCTTGGAAGAGGAGAACTGGACG | TTGTGAACAGAGTTAGCTGGTAAATGTCCTCTTA |
| 2 | CFH EXON 2 | 0.06 | CTGACAGGTTCCTGGTCTGACCAAACATATCCAG | AAGGCACCCAGGCTATCTATAAATGCCGCCCTGGATATAGATCTCTTGG |
| 3 | CFH EXON 3 | 0.05 | cgttttagAAAGGCCCTGTGGACATCCTGGA | GATACTCCTTTTGGTACTTTTACCCTTACAGGAGG |
| 4 | CFH EXON 4 | 0.05 | GCTAGGTGAGATTAATTACCGTGAATGTGACACAGAT | GGATGGACCAATGATATTCCTATATGTGAAGgtagac |
| 5 | CFH EXON 5 | 0.05 | GTCAGTAGTGCAATGGAACCAGATCGGGAATA | CCATTTTGGACAAGCAGTACGGTTTG |
| 6 | CFH EXON 6 | 0.06 | GAAAGAGGAGATGCTGTATGCACTG | AATCTGGATGGCGTCCGTTGCCTTCAT |
| 7 | CFH EXON 8 | 0.05 | CAGGAAGTTACTGGGATCACATTCATTGCACACAAG | ATGGATGGTCGCCAGCAGTACCATGCCTCA |
| 8 | CFH EXON 9 | 0.06 | GTAAATCTATAGACGTTGCCTGCCATCCTGGCTACGC | TCTTCCAAAAGCGCAGACCACAGTTACATGTATGG |
| 9 | CFH EXON 11 | 0.06 | GATGGATGGTCAGCTCAACCCACGTGCATTAGTAA | GTAATTTATTATGTTTGTATTGATTATCCAGATGATACAC |
| 10 | CFH EXON 12 | 0.06 | GATGACATTAGAAATGACATTCTAAATTTTTTATGC | ACTAGAATCTTGTGATATCCCAGTATTTATGAATGCCAGAAC |
| 11 | CFH EXON 13 | 0.09 | CTGCAAACCAGGATTTACAATAGTTGGACCTA | ATTCCGTTCAGTGCTACCACTTTGGATTGTCTC |
| 12 | CFH EXON 14 | 0.07 | GCAAGTACAATCATGTGGTCCACCTCCTGAACTCC | TCAATGGGAATGTTAAGGAAAAAACGAAAGAAGAATATGGA |
| 13 | CFH EXON 15 | 0.07 | CGTGTATTCATGGAGTATGGACCC | AACTTCCCCAGTGTGTGGgtgaga |
| 14 | CFH EXON 16 | 0.06 | CAGTCTGCATAAATGGAAGATGGGATC | CAGAAGTGAACTGCTCAAgtaagctct |
| 15 | CFH EXON 17 | 0.05 | GTGGCACAAATACAATTATGCCCACC | TCCACCTCAGATTCCCAATTCTCACA |
| 16 | CFH EXON 18 | 0.09 | CCATGTTCACAACCACCTCAGATAGAACAC | GGAACCATTAATTCATCCAGGTCTTCAC |
| 17 | CFH EXON 19 | 0.07 | GGGAGAGAAGAAGGATGTGTATAAGGCG | GGTGAGCAAGTGACTTACACTTGTGCAAC |
| 18 | CFHR1_1 | 0.1 | GAACCATCATCAATTTCAAAACCCG | TGTCCTCTTAGTGTTACTCCAAAGaatgttg |
| 19 | CFHR2_1 | 0.1 | CGACATCTTGAGTCCTTATTTGGCAGGTTCCATTGCTGCCA | TGGTGGGTATAAGCCCAGGTTTCCCATCTTTCATGGTCCTG |
| 20 | CFHR3_1 | 0.1 | CAAAAGCGCAGACCACAGTTACATGTAC | GGAGAAAGGCTGGTCTCCTACTCCCAGATGCATC |
| 21 | CFHR4_1 | 0.1 | GAGTAATGGCTACTTGGGAAGATGATCATT | CCATCTCTCTCAATTCAATTTGCCTTTACG |
| 22 | CFHR5_1 | 0.08 | GTACCCAGGCTTAACTCATGTTTCCA | CTGCCCCAGAACTAGAATCAGCCATA |
| 23 | F13B EXON 3 | 0.07 | CTAGCGGAGGGAAGGATGAAGAAGTGGTTCAATGTCTCTCTGAT | GGATGGTCTTCTCAACCAACCTGTAGGAAAGAACATGGTATAAGC |
| 24 | F13B EXON 7 | 0.06 | ATAAAGTGACATATGCATGTAAAAGCGGCTACCTTC | TCCATGGATCGAATGAGATAACTTGTAATCGTC |
| 25 | F13B EXON 12 | 0.08 | CTCCCTCAAGCACTCTGTCTTATCAAGAACCCT | TAAGAACATAGAAATGAATGGCAGAAAGAGGAGTCC |
| 26 | ARMS2 EXON 1 | 0.07 | GCAAGTCTGTCCTCCTCGGTGGTTCCT | GTGTCCTTCATTTCCACTCTGCGAGAGTCTGTGCTGG |
| 27 | AMRS2 EXON 2 | 0.04 | CTTCTGCTCTGTGCAGCTGGTGAAATC | TTTCTCAACCCTTGAGATGCAGCCC |
| 28 | HTRA1 INTRON 1 | 0.05 | gaccccaggacaaataagaggaatgg | gggcataaaggaaggagagaagttc |
| 29 | HTRA1 EXON 2 | 0.06 | gcttttgttctcagGGCAGGAAGATCCCAACAGTTTG | CGCCATAAATATAACTTTATCGCGGACGTGGTGGAG |
| 30 | HTRA1 EXON 3 | 0.08 | CTGATCGTGACAAATGCCCACGTGGTGACCAACAAGCACCGGGT | CAAAGTTGAGCTGAAGAACGGTGCCACTTACGAAGCCAAAATCAAGGATG |
| 31 | HTRA1 EXON 4 | 0.05 | CATCGGAAGCCCGTTTTCCCTTCA | AAACACAGTCACCACCGGGATCGTGA |
| 32 | HTRA1 EXON 5 | 0.04 | gtttaattgcagTATGGAAACTCGGGAG | GCCCGTTAGTAAACCTGgtaaggtctt |
| 33 | HTRA1 EXON 6 | 0.05 | cacgattcagtaagccgtgtccttctt | gcttttcagGACGGTGAAGTGATTGGAA |
| 34 | HTRA1 EXON 7 | 0.04 | GGTATCCGAATGATGTCACTCACGTCCAG | gtgggtaaacaggatgcgtgtctgtgtct |
| 35 | HTRA1 EXON 8 | 0.08 | CAAAGCCAAAGAGCTGAAGGACCGG | CACCGGGACTTCCCAGACGTGATCT |
| 36 | HTRA1 EXON 9 | 0.06 | GATCACAGTGATTCCCGAAGAAATTGACCCATAGG | CAGAGGCATGAGCTGGACTTCATGTTTCCCTC |
|  | Validation Probes |  |  |  |
|  | CFHR2_2 | 0.07 | CACTCTATGTCCAAGGTTGAATGG | TAGCTCAAGAACGCTGTGGAGAGT |
|  | CFHR3_2 | 0.1 | CCCAGCATGTTCATGTCTTTCTAAGTAAC | ACGGACGACAGTCTCAGACTTGTCTAGTTTCTT |
|  | CFHR4_2 | 0.1 | CATGGgagtgcaacttacgatccaagtca | tccctagaagttgtagggcttcaaGTAGC |
|  | CFHR5_2 | 0.06 | CTGAGTTCTTCCTGGGTTCTCCCT | CCTTGCACAGTCTAGAAAAGGCTTCCC |
|  | ARMS2 INTRON 1 | 0.07 | cagcctgcttctcgtccgggttgttagaggagtcatttagaaa | gctgtaccattctttcaatattctcacggctttccagtgctc |

43 probes used to characterize the *CFHR* gene cluster, *CFH, AMRS2*, *HTRA1* and *F13B* genes on chromosome 1q32 and 10q26. Note the standard deviation (STD) of all probes is <10% across 50 samples indicating the reliability of each probe.
