# Supplementary material for: Comprehensive Analysis of Copy Number Variation of Genes at Chromosome 1 and 10 Loci Associated with Late Age Related Macular Degeneration
Source: PLoS One. 2012 Apr 25;7(4):e35255. doi: 10.1371/journal.pone.0035255 (PMC3338825; doi:10.1371/journal.pone.0035255)
Supplement: Table S2 — Real time Quantitative PCR primer sequences. (DOC) [file pone.0035255.s002.doc]

**Table S2: Real time Quantitative PCR primer sequences**

| **Probe** | **5' Forward Primer 3'** | **5' Reverse Primer 3'** |
| --- | --- | --- |
| **CFHR3** | GAGGGTATTCATCAGAATTGGG | AAGCAACTCACAAAGTATATCCTTC |
| **CFHR4** | CAGATGGTGACAGTGAATCTGC | CCCAAGACCAGGATCTTACAG |
| **FOXP2** | TGACATGCCAGCTTATCTGTT T | GAGAAAAGCAATTTTCACAGTCC |

Two primer pairs were used to characterize the *CFHR3-1* and *CFHR1-4* deletions. Included are the primer sequences for the FOXP2 used as the internal control gene.
