# Supplementary material for: Comprehensive Analysis of Copy Number Variation of Genes at Chromosome 1 and 10 Loci Associated with Late Age Related Macular Degeneration
Source: PLoS One. 2012 Apr 25;7(4):e35255. doi: 10.1371/journal.pone.0035255 (PMC3338825; doi:10.1371/journal.pone.0035255)
Supplement: Table S3 — Validation of copy number changes in the CFH gene using locus specific QMPSF. (DOC) [file pone.0035255.s003.doc]

**Table S3: Validation of copy number changes in the *CFH* gene using locus specific QMPSF**

| **Sample ID** | **Exon** | **MLPA^a^** | **QMPSF^b^** |
| --- | --- | --- | --- |
| A-3049 | CFH Exon 18 | 0.64 | 1.09 |
| B-10041 | CFH Exon 18 | 0.68 | 0.98 |
| C-3635 | CFH Exon 18 | 0.65 | 1.00 |
| D-10010 | CFH Exon 18 | 0.67 | 0.86 |
| E-1900 | CFH Exon 18 | 0.63 | 0.84 |
| F-308 | CFH Exon 18 | 0.63 | 1.04 |
| G-10484 | CFH Exon 18 | 0.67 | 0.90 |
| H-10835 | CFH Exon 18 | 0.64 | 0.97 |
| I-1549 | CFH Exon 18 | 0.65 | 0.94 |

Table: ^a^Copy number calculations for MLPA were performed as described in the methods. ^b^Copy number calculations for QMPSF were performed by dividing the peak height of the locus specific peak by the sum of the control probe, then dividing the normalized value by the median of all control samples.
