# Supplementary material for: Comprehensive Analysis of Copy Number Variation of Genes at Chromosome 1 and 10 Loci Associated with Late Age Related Macular Degeneration
Source: PLoS One. 2012 Apr 25;7(4):e35255. doi: 10.1371/journal.pone.0035255 (PMC3338825; doi:10.1371/journal.pone.0035255)
Supplement: Table S4 — Primers sequences for QMPSF analysis. (DOC) [file pone.0035255.s004.doc]

**Table S4**: Primers sequences for QMPSF analysis

| **Primer** | **5' Forward Primer 3'** | **5' Reverse Primer 3'** |
| --- | --- | --- |
| CFH EXON 18 | TCCATGTTCACAACCACCTC | GATATCCTGAAACCACCCTCAC |
| HMBS Control | TAGACGGCTCAGATAGCATACAAG | ATGCCTACCAACTGTGGGTCA |
